# Phosphorylation of NF-κBp65 drives inflammation-mediated hepatocellular carcinogenesis and is a novel therapeutic target

**DOI:** 10.1186/s13046-021-02062-x

**Published:** 2021-08-11

**Authors:** Xuan Xu, Yiming Lei, Lingjun Chen, Haoxiong Zhou, Huiling Liu, Jie Jiang, Yidong Yang, Bin Wu

**Affiliations:** 1 Department of Gastroenterology, the Third Affiliated Hospital of Sun Yat-Sen University, Guangzhou, 510630 Guangdong Province China; 2 Guangdong Provincial Key Laboratory of Liver Disease Research, Guangzhou, 510630 Guangdong Province China

**Keywords:** Hepatocellular carcinoma, Inflammation, NF-κBp65, Phosphorylation, β-arrestin1

## Abstract

**Background:**

Nuclear factor­κB (NF-κB) plays a vital role in hepatocellular carcinoma (HCC). β-arrestin1 (ARRB1) has been proved to enhance the activity of NF-κBp65, and our previous study indicated that ARRB1 promotes hepatocellular carcinogenesis and development of HCC. However, it remains unknown whether p65 is involved in hepatocellular carcinogenesis through the ARRB1-mediated pathway.

**Methods:**

The levels of NF-κBp65 and NF-κBp65 phosphorylation (p-p65) were assessed in including normal liver, primary HCC and paired paracancerous tissues. Liver-specific p65 knockout mice were used to examine the role of p65 and p-p65 in hepatocarcinogenesis. The mechanism of NF-κBp65 and p-p65 in hepatocarcinogenesis via ARRB1 was also studied both in vitro and in vivo.

**Results:**

Phosphorylation of NF-κBp65 was markedly upregulated in inflammation-related HCC patients and was significantly increased in mouse hepatic inflammation models, which were induced by tetrachloromethane (CCl_4_), diethylnitrosamine (DEN), TNF-α, as well as DEN-induced HCC. Hepatocyte-specific *p65*-deficient mice markedly decreased in the HCC incidence and size of tumours by the repressing of the proliferation of malignant cells in a DEN-induced HCC model. Furthermore, ARRB1 directly bounds p65 to promote the phosphorylation of NF-κBp65 at ser536, resulted in cell malignant proliferation through GSK3β/mTOR signalling.

**Conclusion:**

The data demonstrated that phosphorylation of NF-κBp65 drives hepatocellular carcinogenesis in response to inflammation-mediated ARRB1, and that inhibition of the phosphorylation of NF-κBp65 restrains the hepatocellular carcinogenesis. The results indicate that phosphorylation of NF-κBp65 is a novel therapeutic target for HCC.

**Supplementary Information:**

The online version contains supplementary material available at 10.1186/s13046-021-02062-x.

## Background

Hepatocellular carcinoma (HCC), the most common neoplasm among all primary liver cancers, is currently the sixth most common malignancy and the second leading cause of cancer-related death worldwide [1, 2]. Numerous results have demonstrated that chronic inflammation is connected to hepatocarcinogenesis, and chronic inflammation caused by persistent infections with hepatitis B virus (HBV) and hepatitis C virus (HCV) or nonalcoholic steatohepatitis (NASH) can increase cancer risk [3].

Nuclear factor­κB (NF-κB) is activated in response to different infectious agents and inflammatory cytokines, and it plays a central role in inflammation control and immunosuppression via several mechanisms [4]. The mammalian NF-κB family consists of five transcription factors: p65 (also known as RelA), RelB, c-Rel, p105/p50 (NF-κB1), and p100/p52 (NF-κB2). Although p65, RelB and c-Rel are final proteins, p50 and p52 are produced by proteolytic processing of p105 and p100 [5]. All transcription factors are activated only when they form heterodimers or homodimers and induce nuclear translocation after a series of stimulations, among which RELA-p50 is most commonly detected and takes charge of the most transcriptional activity [4]. Inhibitor of κB (IκB) proteins can inhibit the activation of NF-κB dimers by interacting with the nuclear localization signal (NLS) of the highly conserved Rel homology domain (RHD), inducing a shift from the cytoplasm to the nucleus. However, IκBs are regulated by the IκB kinase (IKK) complex, which is composed of two catalytic subunits, IKKα and IKKβ, and a regulatory component called IKKγ or NF-κB essential modulator (NEMO). Many stimuli activate NF-κB through IKK-dependent phosphorylation, causing degradation of IκBs [4, 6, 7]. Previous studies have established IKKβ-deficient mice and demonstrated that inactivation of the IKK-NF-κB pathway can attenuate the promotion of a colitis-associated cancer model [8]. NF-κB was found to be activated in human HCC, especially hepatitis-related human HCC [9]. The activation of NF-κB in solid malignancies was due to increased activation of IKK cytokines, including tumour necrosis factor (TNF) and IL­1 [10, 11].

G protein-coupled receptors (GPCRs), the largest family of membrane proteins, play critical roles in mediating physiological responses and activation of crosstalk signalling related to human cancer [12]. According to a previous view, GPCR signalling couples to the G protein and triggers canonical transduction cascades, while β-arrestin1 (ARRB1) can be recruited and results in GPCR desensitization [13]. The dominant role of ARRB1 is to dampen the activity of NF-κB by binding to IκBs [14, 15]. Our previous study also found that ARRB1 is involved in hepatocarcinogenesis via the PI3K/Akt pathway [16]. Certain studies have confirmed the role of ARRB1 in tumorigenesis, including nicotine-induced carcinogenesis in the lungs by nuclear translocation of ARRB1 and LPA-induced breast cancer through ARRB1-mediated cell migration and invasion [17, 18].

Akt, also known as protein kinase B, is a serine/threonine protein kinase that mediates several cellular functions, including cell growth and proliferation, through phosphatidylinositol 3-kinase (PI3K). Akt is frequently activated in human solid tumours, including HCC [16]. Glycogen synthase kinase 3β (GSK3β), a serine/threonine protein kinase, is found to induce the phosphorylation of diverse substrates and to be one of the first identified substrates of kinase Akt. Inhibition of GSK3β caused by phosphorylation of the Ser9 site can deactivate GSK3β through Akt signalling and stabilize downstream target proteins by inhibiting ubiquitin/proteasome-mediated degradation [19]. Several reports have also found that GSK3β plays a critical role in the promotion of HCC and can increase proteins related to cell growth through phosphorylation of mTOR [20].

Previous studies have indicated that both ARRB1 and the activated NF-κB pathway are related to inflammation-related HCC. However, whether activated NF-κB signalling is involved in ARRB1-mediated hepatocarcinogenesis remains unknown. In our study, these data showed that phosphorylation of NF-κBp65 is induced in HCC patients and that enhanced ARRB1 directly induces the phosphorylation of p65 by binding. Together, our data suggested that phosphorylation of p65 is related to ARRB1-mediated hepatocellular carcinogenesis via GSK3β/mTOR and that inhibitors of NF-κBp65 could be therapeutic targets for HCC.

## Materials and methods

### Clinical samples, tissue microarray and microarray experiment

This study was approved by the Clinical Research Ethics Committee of the Third Affiliated Hospital of Sun Yat-Sen University. All of the patients were informed of the use of their data before surgery was performed. Specimens of primary HCC and paired paracancerous tissues were obtained from HCC patients who received curative surgery at the Third Affiliated Hospital of Sun Yat-Sen University. Normal liver tissues were obtained from adjacent tissues in haemangioma patients who received hepatectomy. Samples were used for tissue microarray. Whole blood (3 ml) from healthy volunteers and HBV-infected HCC patients was collected by venepuncture. After static duration for 30 min at room temperature, samples were centrifuged for 15 min at 2000 g. The serum was obtained and stored at - 80 °C for further determination of total protein. The gene expression experiments were performed as described previously [16].

### Cell culture and establishment of cell lines

HepG2 and Hep3B cells were obtained from the American Type Culture Collection (ATCC, Manassas, VA, USA). HepG2, Hep3B and HepG2.2.15 cells were cultured in Dulbecco’s modified Eagle’s medium (DMEM) (Gibco BRL, Rockville, MD, USA) supplemented with 10% foetal bovine serum (FBS) (Gibco BRL) in an incubator with 5% CO_2_ at 37 °C. LO2 cells were cultured in RPMI 1640 medium (Gibco BRL) with 10% FBS. For activator intervention, LO2, HepG2, Hep3B and HepG2.2.15 cells were treated with TNF-α (40 ng/ml) purchased from Proteintech Group (Rosemont, IL, USA) for 4 h. For the NF-κB inhibitor, 10 μM Bay 11-7082 (Sigma, St Louis, MO, USA) was added to the cell lines 6 h after 2 h of pretreatment with TNF-α (40 ng/ml). The lentiviral vector encoding the human *ARRB1* gene was purchased from GeneChem (Shanghai, China) Corp. We transfected the cells with the ARRB1 lentivirus according to the instructions and obtained stable ARRB1-expressing LO2, HepG2, Hep3B and HepG2.2.15 cell lines by puromycin selection (2 μg/ml). We also purchased p65 wild type (WT) and p65 mutant (S536A), of which serine 536 could not be phosphorylated as inactive p-p65, while p65 mutant (S536E) could mimic phospho-p65 as active p-p65.

### Mice

The study was approved by the Institutional Animal Care and Use Committee of the Third Affiliated Hospital of Sun Yat-Sen University. Hepatocyte-specific *p65* knockout (*L-p65* KO) (p65^f/f^, Alb-cre^+/−^) mice were obtained by crossing floxed-p65 littermate (p65^f/f^) mice with Alb-cre^+/−^ mice as described previously [21]. Floxed-p65 littermates (p65^f/f^) were used as wild-type (WT) mice. WT mice and *L-p65*-KO mice were produced by heterozygote intercrosses on a C57BL/6 background. The mice were housed in microisolator cages under a 12/12-h dark-light cycle (lights on at 8:00 a.m.) with food and water ad libitum. The sedative consisted of xylazine (15 mg/kg), and ketamine (50 mg/kg) was administered intraperitoneally as anaesthesia. Carbon dioxide inhalation was used as the method of euthanasia. Animal research protocols were authorized by the Institutional Animal Care and Use Committee of the Third Affiliated Hospital of Sun Yat-Sen University.

### Treatment of mice

All of the studies were conducted in at least 6 male mice in each group following the protocol described previously [16, 22]. For tetrachloromethane (CCl_4_)-induced mouse liver cirrhosis models, 6- to 8-week-old mice were intraperitoneally injected with CCl_4_ (Sigma, St Louis, MO, USA) at 5 mg/kg twice per week. CCl_4_ was dissolved in olive oil (Macklin Biochemical, Shanghai, China) at a ratio of 1:4, and vehicle mice were injected with olive oil. The mice were sacrificed 8 weeks after injection. For diethylnitrosamine (DEN)-induced hepatitis models, 6- to 8-week-old mice received an intraperitoneal injection of DEN (100 mg/kg) (Sigma, St Louis, MO, USA). The mice were sacrificed 10 days after injection, and vehicle mice were injected with physiological saline at an equivalent volume. For TNF-α-induced acute liver inflammation models, 6- to 8-week-old mice were intraperitoneally injected with TNF-α dissolved in cell culture medium (DMEM) 6 h before being sacrificed. For the DEN-induced HCC model, 14-day-old mice were treated with an intraperitoneal injection of DEN (15 mg/kg). The tumour incidence was analysed 9 months after injection, and the tumour nodules were measured with Vernier callipers.

### MRI scans in DEN-induced HCC mouse model

Nine months after DEN injection, mice were anaesthetized with isoflurane at room temperature. The mice were placed in a 7.0 T MRI tomograph (PharmaScan70/16, Bruker BioSpin, Ettlingen, Germany) with continuous narcosis. The respiratory and heart rates of the mice were monitored during MRI imaging.

### Protein extraction and western blotting

Total protein extracts from normal tissues, HCC liver tissues and cell lysates were obtained by RIPA buffer treatment and examined via western blotting as described previously [16]. Blots were imaged using ChemiDoc imaging system (Bio-rad, Hercules, CA, USA). Serum proteins were diluted 50 times before being subjected to western blotting. Antibodies against p65 (1:1000, 8242, CST, Danvers, MA, USA), p-p65 (1:1000, 3033, CST), PCNA (1:2000, 13,110, CST), ARRB1 (1:1000, 12,697, CST), TNF-α (1:1000, 6945, CST), PI3K (1:1000, 4249, CST), Akt (1:1000, 9272, CST), p-Akt (1:1000, 4060, CST), GSK3β (1:1000, 12,456, CST), p-GSK3β (1:1000, 9323, CST), mTOR (1:1000, 2983, CST), p-mTOR (1:1000, 5536, CST) and β-actin (1:3000, A5441, Sigma-Aldrich, St Louis, MO, USA) were used as primary antibodies. Goat anti-mouse (1:5000, 7076, CST) or goat anti-rabbit (1:5000, 7074, CST) HRP-linked antibodies were used as secondary antibodies. After quantifying the densitometry of each target protein and β-actin, the results were expressed as normalized ratios.

### Co-immunoprecipitation assay (co-IP assay)

For the co-IP assay, ARRB1-overexpressing HepG2 cells and TNF-α pretreatment HepG2 cells were used to obtain protein lysates. Protein A-Magnetic Beads (100 μl) were conjugated with antibodies against GFP, ARRB1 and IgG (CST) at room temperature for 1 h. The cell lysates were cleared, and preconjugated magnetic beads were added to the supernatant to pull the immune complexes by spinning them at 4 °C overnight. The beads were washed three times with 0.1% PBS, soaked in lysis buffer and heated for 10 min at 70 °C. The supernatant was subjected to western blotting assay as described previously with ARRB1-, p65- and p-p65-specific antibodies (CST).

### Immunohistological staining, immunofluorescence staining and EdU cell proliferation assay

Liver sections were subjected to H&E staining and immunohistochemical (IHC) staining according to the protocol in our previous study [16]. Immunofluorescence (IF) staining of HCC cell lines was also performed as previously described [16]. For IHC staining, antibodies against p65 (1:200, 8242, CST), p-p65 (1:200, SAB4300009, Sigma), NF-κB1 (1:100, A6667, Abclonal, Woburn, MA, USA), p-NF-κB1 (1:100, AP0417, Abclonal), NF-κB2 (1:100, A3108, Abclonal), p-NF-κB2 (1:100, AP0418, Abclonal), RELB (1:100, A0519, Abclonal), p-RELB (1:100, AP0240, Abclonal), c-Rel (1:100, GTX113264, GeneTex, Irvine, CA, USA), p-c-Rel (1:100, ab30624, Abcam, Cambridge, MA, USA), Ki67 (1:400, ab15580, Abcam), ARRB1 (1:100, Abcam ab32099), p-Akt (1:100, CST), and p-GSK3β (1:200, CST) were used as primary antibodies. The EnVision method (EnVision Kit/Alakline Phosphatase detection system, DAKO, Glostrup, Denmark) was used, and haematoxylin was used for liver section counterstaining. For cell IF staining, after fixation, permeabilization and incubation with specific primary antibodies against p65, fluorescence-conjugated secondary antibodies Alexa 594 (Molecular Probes, Eugene, OR, USA) and DAPI (Molecular Probes) were performed. Images were analysed using fluorescence microscopy. An EdU DNA Cell Proliferation Kit (RiboBio, Guangzhou, Guangdong province, China) was used to assess cell proliferation and was performed according to the manufacturer’s protocol. The nuclei of proliferative cells were dyed red. For double staining of p65 and EdU, IF staining of cells was performed before the EdU assay, and the EdU index was calculated as follows: the number of red nuclei / the number of total cells.

### Cell viability and colony formation assay

To evaluate cell growth, a CCK-8 assay (Dojindo Laboratories, Kumamoto, Japan) was used. Cells (1 × 10^3^ cells) were seeded in 96-well plates per well with 100 μl of complete medium overnight. Following Bay 11-7082 inhibitor incubation for 24 to 96 h, Cell Counting Kit-8 (CCK-8) solution (10 μl) was then added to each well and incubated for 2 h at 37 °C before measuring OD values at an absorbance of 450 nm at each time point (BioTek, Winooski, VT, USA). For the colony formation assay, cells (1 × 10^3^ cells) were seeded in 6-well plates per well. Following 14 days of incubation with a low dose of Bay 11-7082 inhibitor, the cells were washed, fixed and stained with crystal violet. All of the cells were seeded in triplicate wells, and all of the experiments were performed three times.

### Xenograft tumours

Xenograft tumours were established in 4-week-old male Balb/c nude mice purchased from Vital River Laboratory Animal Technology Co., Ltd. (Beijing, China). Approximately 1 × 10^6^ Hep3B cells were injected subcutaneously into nude mice, and tumour growth was recorded at each time point. The tumour volume was calculated by the formula (length×width^2^/2), and the tumour was supposed to be established when the length and width were both more than 5 mm. The nude mice were equally divided into two groups according to the volume and treated with an intraperitoneal injection of PDTC (an NF-κB inhibitor, 120 mg/kg) (Sigma) once per day for 12 days, and tumour growth was recorded every 3 days after the first injection of inhibitor.

### Real-time PCR analysis

Total RNA was extracted using a Tissue RNA Purification Kit RN002 (ES Science, Shanghai, China) and transcribed into cDNA using a High Capacity cDNA Kit FSQ101 (TOYOBO, Osaka, Japan). For quantitative real-time polymerase chain reaction (PCR) analysis, aliquots of cDNA were amplified using gene-specific primers and ChamQ SYBR qPCR Master Mix (Vazyme, Nanjing, Jiangsu province, China) in a real-time PCR system (Bio-Rad). Each sample was tested in triplicate. RNA was amplified using the following primers: Human *p65* exon sense 5′- ATGTGGAGATCATTGAGCAGC-3′, and human *p65* exon antisense 5′- CCTGGTCCTGTGTAGCCATT-3′; mouse *p65* exon sense 5′-TGCGATTCCGCTATAAATGCG-3′, and mouse *p65* exon antisense 5′-ACAAGTTCATGTGGATGAGGC-3′; mouse *TNF-α* exon sense 5′-CTTCATCACCTATCCCTCGAC-3′ and mouse *TNF-α* exon antisense 5′-CTGGCTATTTGCTTCTTGTCCT-3′. The expression of *β-actin* was quantified as the internal control using the sense primer 5′-GTCTTCCCCTCCATCGTG-3′ and the antisense primer 5′-AGGGTGAGGATGCCTCTCTT-3′ for human samples, and the sense primer 5′-GGCTGTATTCCCCTCCATCG-3′ and the antisense primer 5′-CCAGTTGGTAACAATGCCATGT-3′ for mouse samples.

### Analysis of p65 and p-p65 translocation

To analyse p65 and p-p65 translocation into the nucleus, samples of cell lines or an aliquot of liver tumour cells isolated from the DEN-induced HCC mouse model was collected. Then, nuclear and cytoplasmic fractions were isolated using the Nuclear Extract Kit (Active Motif, Carlsbad, CA, USA) according to the manufacturer’s protocol. Aliquots of both fractions were mixed with equal volumes of 2× Laemmli sample buffer and analysed by western blotting for p65 and p-p65.

### *P65* and *ARRB1* reporter assays

Two hundred ninety-three T cells (2 × 10^4^/well) were plated in 24-well plates and transfected with the expression plasmid pcDNA3.1- p65 (WT)-HA or pcDNA3.1- p65 (S536A)-HA or pcDNA3.1- p65 (S536E)-HA or the vector pcDNA3.1-HA (400 ng/well) using Lipofectamine 3000 (L3000, Invitrogen, Carlsbad, CA, USA). For each transfection, 200 ng of the pGL4.1-p65 luciferase reporter plasmid or the pGL4.1-ARRB1 luciferase reporter plasmid, combined with 5 ng of the Renilla plasmid as a control, was used. Luciferase activities were detected by the Dual-Luciferase Reporter Assay System (E1910, Promega, Fitchburg, WI, USA) according to the manufacturer’s instructions with a Lumat LB 9507 luminometer (Berthold, Nashua, NH, USA).

### Statistical analyses

The results are presented as the means ± standard deviation (SD). The significance of variables of two groups was determined by the independent-samples t-test. The protein levels of p-p65 in normal liver, HCC and matched paracancerous tissues of humans were compared using one-way ANOVA. The χ^2^-test was used to analyse the Ki67 rate differences among mouse normal liver tissues and paired paracancerous and HCC samples. Values of *P* < 0.05 were considered statistically significant. All of the data analyses were performed using GraphPad Prism Version software, version 7.0, and SPSS software, version 22.0.

## Results

### Expression of NF-κB family members and phosphorylation of p65 in HCC patients

**Fig. 1:**
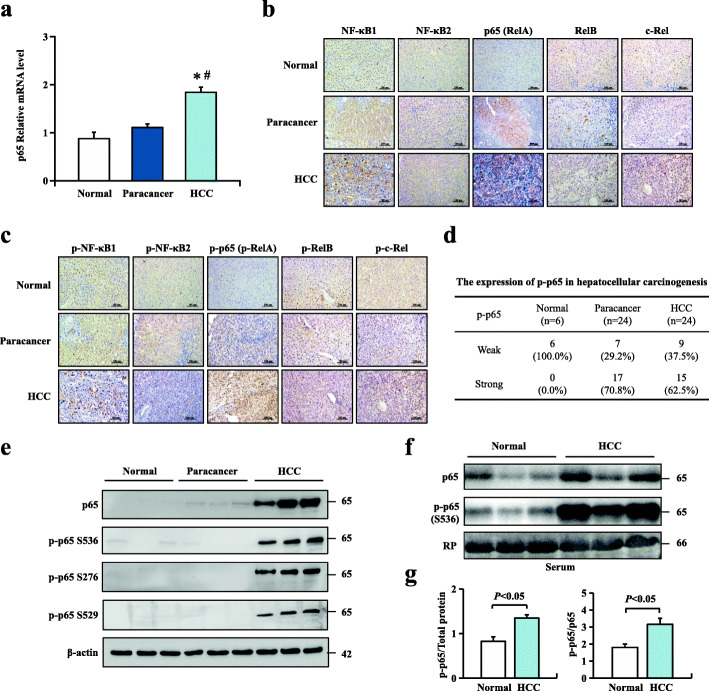
Expression of NF-κB family members and phosphorylation of p65 in HCC patients. **a** p65 mRNA levels in normal human liver, HCC and paracancerous tissue were analysed by real-time PCR. Values are mean ± s.d. (*n* = 6 for normal liver, *n* = 10 for HCC and matched paracancerous tissues); *P < 0.05 compared with normal liver tissues, ^#^*P* < 0.05 compared with paracancerous tissues by one-way ANOVA. **b** Immunohistochemical analysis of NF-κB subunits from normal human liver, HCC and matched paracancerous tissues. **c** Immunohistochemical analysis of phospho-NF-κB subunits in normal human liver, HCC and matched paracancerous tissues. **d** Immunohistochemistry staining was performed to confirm the expression of p-p65 (p-RelA) in 6 normal liver tissue samples and 24 paired HCC samples with matched paracancerous tissues. **e** p65 and p65 S536, p65 S276, and p65 S529 protein expression levels in normal human liver tissue, HCC and paracancerous tissue using western blotting assay. **f**, **g** p65 and p-p65 protein expression in the blood serum of healthy volunteers and HCC patients. The bottom panel shows red Ponceau (RP) staining of the PVDF membrane after transfer as a control of loading. The position and size in kilodaltons of molecular mass markers are indicated on the right. Values are mean ± SD (*n* = 6 in each group). *P* < 0.05 using Student’s *t*-test

In recent years, the molecular mechanisms between inflammation and tumorigenesis have been largely evaluated. As a key regulator of inflammation and immune responses, the NF-κB pathway can act as a promoter or tumour suppresser [23]. To assess the relationship between NF-κBp65 and human HCC, mRNA level results revealed that p65 was significantly upregulated in HCC compared with normal liver tissues and paracancerous tissues (Fig. 1a). Immunohistochemistry was performed to detect the protein levels of five cellular DNA-binding subunits of NF-κB in 6 normal liver tissues and matched HCC with adjacent paracancerous tissues. Significant differences in p65 expression among normal tissues, paracancerous tissues and HCC tissues were found. Protein levels revealed that p65 was significantly upregulated in HCC compared with normal liver tissues and paracancerous tissues (Fig. 1b). Furthermore, the protein levels of the phosphorylated forms of five subunits of NF-κB were also detected by immunohistochemistry staining. Consistently, upregulated p-p65 expression levels were found in both paracancerous (70.8%) and HCC tissues (62.5%) compared with normal tissues (Fig. 1c, d). Arrested p-p65 staining, predominantly in the nucleus, and upregulated p-p65 expression at Ser536, Ser276 or Ser529 residues were observed in HCC liver tissues (Fig. 1c, e). We then examined the serum protein levels of p65 and p-p65 in 6 healthy volunteers and 6 HBV-infected HCC patients using western blotting. The results showed that p-p65 expression was significantly upregulated in the serum of HCC patients, which could be an effective biomarker of HCC patients (Fig. 1f, g). These results indicated that p65, as well as phosphorylation of p65, might play a critical role in inflammation-related hepatocellular carcinogenesis. Previous studies have demonstrated that activation of the NF-κB pathway can promote HCC progression and that the classical NF-κB pathway mainly targets p50-p65 heterodimers. We then mainly focused on the clinical significance of p65 and p-p65 in HCC patients.

### Deficiency of hepatocyte p65 inhibited hepatocellular carcinogenesis and cancer growth in mice

**Fig. 2:**
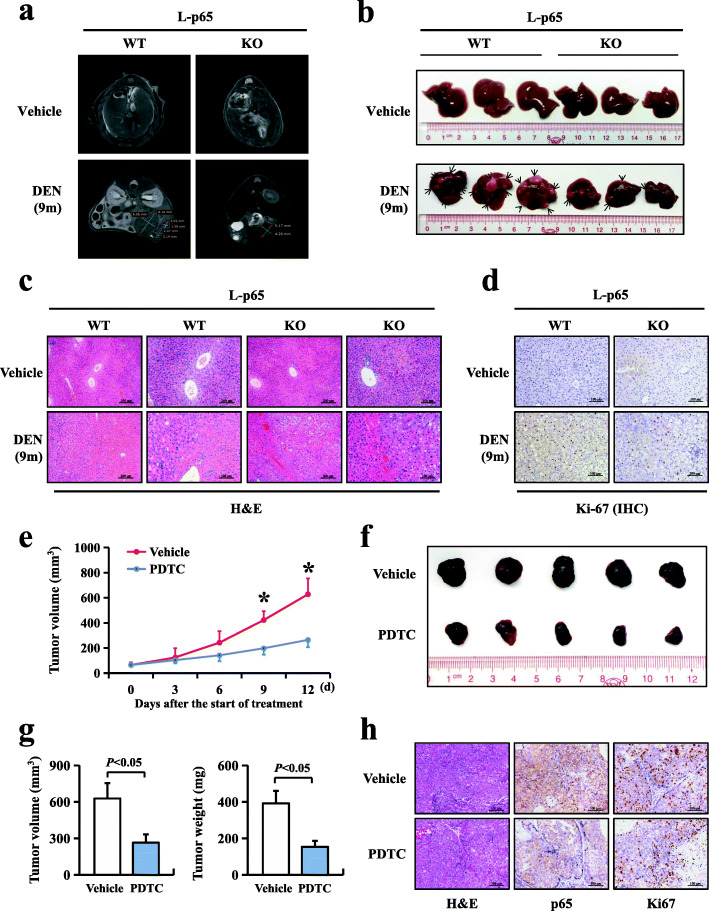
Deficiency of hepatocyte p65 inhibited hepatocellular carcinogenesis and cancer growth in mice. L-p65 wild-type (WT) and L-p65 knockout (KO) mice were treated with a single DEN (15 mg/kg) to induce mouse hepatocellular carcinoma. **a** MRI images of vehicle- and DEN-induced liver tumours. **b** Photographs of vehicle- and DEN-induced liver tumours at 9 months. **c** Representative H&E staining of DEN-induced liver tumours in WT and L-p65-KO mice. **d** Ki67 staining of a DEN-induced liver tumour. **e** The growth curve of xenograft tumours of Hep3B cells subjected to vehicle (normal saline) or PDTC (an inhibitor of IκB). Data are expressed as the mean ± SD (*n* = 5 in each group); *P < 0.05 using Student’s *t*-test. **f** Xenograft tumours of Hep3B cells subjected to vehicle (top) or PDTC (bottom) at the end of the experiment. **g** Xenograft tumour volumes and tumour weights from different treatment groups at the end of the experiment. **h** Representative H&E, p65 and Ki67 staining of xenograft tumours in the Hep3B vehicle and PTDC groups. Data were expressed as the mean ± SD (*n* = 5 in each group). *P* < 0.05 using Student’s *t*-test

Our data showed that p65 expression was increased in HBV-infected HCC tissue in humans (Fig. 1). However, the relationship between p65 and hepatocarcinogenesis still requires further study. Here, to evaluate the effect of p65 on hepatocarcinogenesis, we established a hepatocyte-specific deletion of the *RELA* gene in mice by crossing floxed-p65 littermates (p65^f/f^) with Alb-cre^+/−^ mice, which consequentially lacked the expression of p65 and p-p65. A single intraperitoneal injection of DEN (15 mg/kg) in 14-day-old male mice led to effective HCC production. *L-p65* knockout (KO) (p65^f/f^, Alb-cre^+/−^) male mice and wild-type (WT) (p65^f/f^) male mice were treated with a single injection of DEN. Tumour incidence and size were compared 9 months after injection. Hepatocellular carcinomas developed in all mice. However, the hepatocellular carcinoma incidence was reduced in *L-p65* KO mice compared with WT mice (Fig. 2a, b). No significant difference was found in the serum AFP levels of WT and *L-p65* KO mice since AFP is an early biomarker of HCC (Figure S1a). The percentage of liver weight versus body weight in *L-p65* KO mice was decreased by 1.6 fold compared with WT mice (4.32 ± 1.22 vs. 7.14 ± 2.69) (Figure S1b). The maximal tumour size and average tumour numbers were reduced significantly in *L-p65* KO mice compared with WT mice (Figure S1c, d). Histopathological analysis was used to confirm mouse hepatocellular cancer (Fig. 2c). Ki67 staining was performed to analyse the proliferation of liver tumours, showing that the number of Ki67-positive cells was reduced in *L-p65* KO mice compared with WT mice (Fig. 2d). Furthermore, a tumorigenicity experiment in Balb/c nude mice showed that inhibition of p65 significantly reduced xenograft tumour growth in vivo (Fig. 2e-h). These results demonstrated that deficiency of p65 and p-p65 suppresses DEN-induced hepatocarcinogenesis by downregulating hepatic compensatory proliferation.

### Inflammation-induced NF-κBp65 phosphorylation is involved in hepatocellular carcinogenesis

**Fig. 3:**
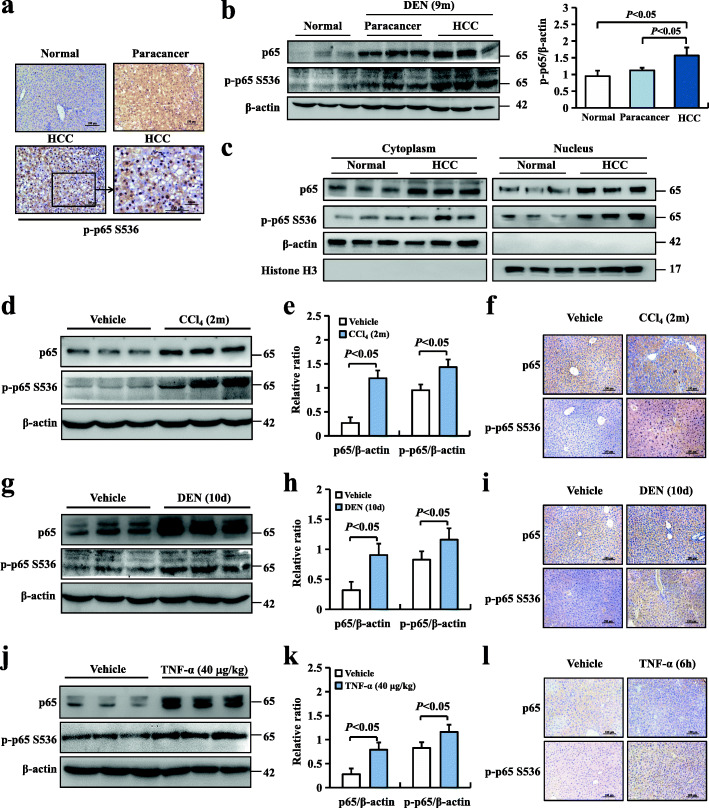
Inflammation-induced NF-κBp65 phosphorylation is involved in hepatocellular carcinogenesis. DEN-induced mouse hepatocellular carcinomas were intraperitoneally injected after either physiological saline (vehicle) or a single DEN (15 mg/kg), and the mouse livers were harvested at 9 months after injection. Normal liver tissues were obtained from vehicle mouse livers, HCC tissues from 9-month DEN-induced mouse liver tumours and paracancerous tissues from adjacent noncancerous tissues. **a** Representative images of p-p65 staining in DEN-induced mouse HCC models. **b** p65 and p-p65 protein expressions in mouse livers were determined using western blotting. **c** p65 was analysed in the nuclear and cytoplasmic fractions of normal liver cells isolated from normal control mice and liver tumour cells isolated 9 months after DEN injection. β-actin and histone H3 were used as the controls for loading and fractionation. **d**, **e** p65 and p-p65 protein expressions in mouse livers were determined by western blotting after either physiological saline (vehicle) or tetrachloromethane (CCl_4_) (15 mg/kg) intraperitoneal injection twice per week for 2 months. **f** Representative images of p65 (top) and p-p65 (bottom) staining in CCl_4_-induced liver cirrhosis. **g**, **h** p65 and p-p65 protein expressions in mouse livers after either physiological saline (vehicle) or 100 mg/kg diethylnitrosamine (DEN) intraperitoneal injection for 10 days were determined using western blotting. **i** Representative images of p65 (top) and p-p65 (bottom) staining in DEN-induced liver inflammation. **j**, **k** p65 and p-p65 protein expressions in mouse livers were determined using western blotting after intraperitoneal TNF-α (40 μg/kg) injection for 6 h. **l** Representative images of p65 (top) and p-p65 (bottom) staining in TNF-α-induced liver inflammation. Values are mean ± SD (*n* = 6 in each group). *P* < 0.05 using Student’s *t*-test

Our data showed that phosphorylation of p65 was increased in HBV-infected HCC and adjacent tissues in humans. However, the role of p-p65 induced by inflammation in carcinogenesis in mice remains unclear. To determine the potential role of p65 phosphorylation in chemical tumorigenicity, we analysed mouse livers 9 months after DEN treatment. All male mice efficiently developed hepatocellular carcinoma by 9 months. Immunohistochemistry was used to determine the phospho-p65 expression of liver tumour samples, and we found that p-p65 was remarkably assembled in the nuclei of hepatocytes (Fig. 3a), and the expression of p-p65 in mouse tumours was increased compared with that in control mouse livers using a western blotting assay (Fig. 3b). To further confirm the translocation of p65 in the DEN-induced HCC mouse model, nuclear and cytoplasmic fractions were isolated. Increased p65 nuclear translocation in DEN-induced mouse HCC models was observed (Fig. 3c). The analyses of tumour cell proliferation were confirmed by Ki67 staining and immunoblotting of PCNA (Figure S2a, b). The Ki67 index and PCNA index showed that cell proliferation was increased in HCC (Figure S2c). Previous data have shown that NF-κBp65-driven inflammation is closely related to hepatocellular carcinoma (HCC), but evidence has shown a doubled-edged effect among numerous mouse models.

We then established several acute inflammation mouse models by tetrachloromethane (CCl_4_, a drug with marked hepatotoxicity), diethylnitrosamine (DEN, a drug that promotes hepatocarcinogenesis by DNA damage) and TNF-α (a potent activator of NF-κB) [10]. Western blotting showed that the protein levels of both p65 and p-p65 were significantly increased in liver tissues after intraperitoneal injection of CCl_4_ for 2 months (Fig. 3d, e), DEN for 10 days (Fig. 3g, h) and TNF-α for 6 h (Fig. 3j, k). Moreover, real-time PCR analysis was performed to measure the mRNA levels of *p65* and *TNF-α* in the aforementioned acute inflammatory mouse models, and elevated *p65* and *TNF-α* mRNA levels were observed (Figure S3). Simultaneously, p-p65 levels were apparently upregulated in the nuclei of hepatocytes after CCl_4_ (Fig. 3f), DEN (Fig. 3i) and TNF-α treatment (Fig. 3l), as determined by immunohistochemistry, indicating increased translocation of p65 into the nucleus. These data suggested that p65 is activated and that the translocation of phosphorylated p65 into the nucleus is markedly increased by CCl_4_-, DEN- or TNF-α-induced hepatic inflammation.

### Inhibiting NF-κBp65 phosphorylation depressed hepatocellular proliferation

**Fig. 4:**
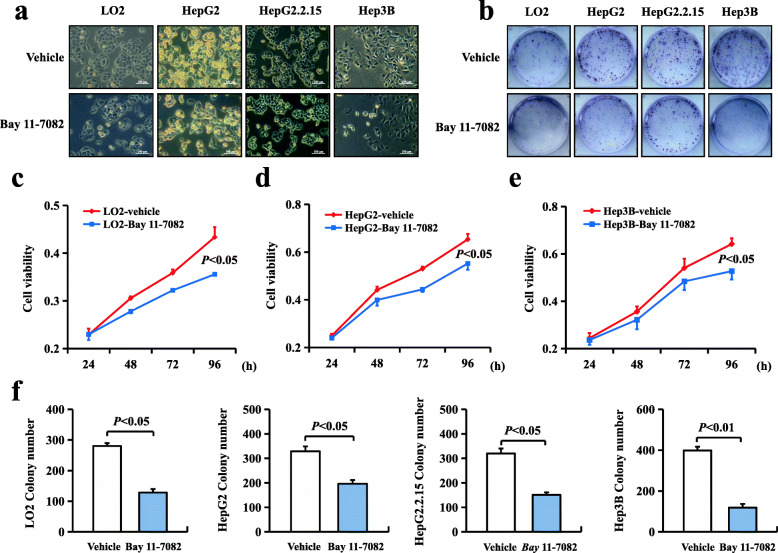
Inhibition of NF-κBp65 phosphorylation depressed hepatocellular proliferation. **a** Morphological changes that occurred when LO2, HepG2, HepG2.2.15 and Hep3B cells were treated with Bay 11-7082 (an inhibitor of IκB) and observed under bright field microscopy. Bay 11-7082 can inhibit the proliferation of cells. **b** Colony formation was suppressed in LO2, HepG2, HepG2.2.15 and Hep3B cell lines in the presence of Bay 11-7082. **c**, **d**, **e** Cell growth curves after Bay 11-7082 treatment. **f** The relative colony formation numbers of Bay 11-7082-treated cells are shown. All values are the means ± SD of three separate experiments and were repeated three times. *P* < 0.05 using Student’s *t*-test

The negative effect of p-p65 deficiency on DEN-induced hepatocarcinogenesis was caused by downregulating proliferation in mice. However, the role of p-p65 in the malignant phenotype of HCC cell lines awaits further study. To evaluate the effect of p-p65, HCC cell lines, including LO2, HepG2, HepG2.2.15 and Hep3B cells, were treated with Bay 11-7082 (an inhibitor of IκB) (Fig. 4a). Bay 11-7082 inhibited cell growth and colony formation (Fig. 4b) among the four HCC cell lines after treatment (Fig. 4c-f). These data indicated that the inhibition of p65 phosphorylation depressed hepatocellular proliferation in human HCC cell lines.

### Phosphorylation of NF-κBp65 Ser536, but not Ser276 and Ser529, was upregulated by inflammation-mediated ARRB1

**Fig. 5:**
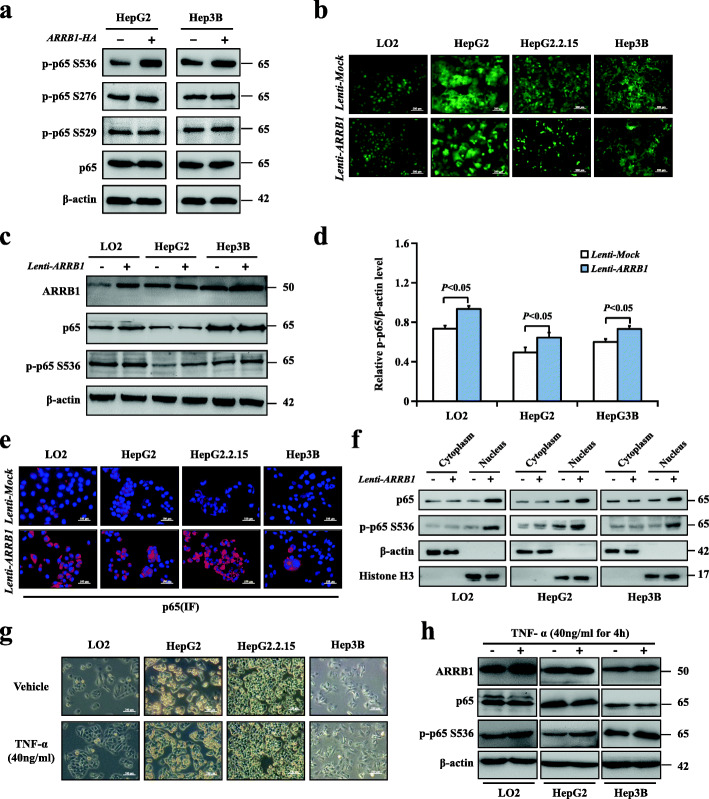
Phosphorylation of NF-κBp65 Ser536, but not Ser276 and Ser529, was upregulated by inflammation-mediated ARRB1. **a** ARRB1 transfection increased the expression of p65 Ser536, but not p65 Ser276 and Ser529, in HepG2 and HepG3B cell lines. **b** Cells were infected with ARRB1 or control lentivirus in LO2, HepG2, HepG2.2.15, and Hep3B cell lines. Cells stably expressed ARBB1 after transfection and selection by puromycin. **c**, **d** ARRB1 transfection increased the expression of p-p65, but not p65, in LO2, HepG2 and Hep3B cell lines. **e** Fluorescence images showed induced nuclear p65 expression in LO2, HepG2, HepG2.2.15, and Hep3B cell lines following transfection of ARRB1 using the immunofluorescence assay. **f** p65 was analysed by western blotting in the nuclear and cytoplasmic fractions isolated from LO2, HepG2, and Hep3B cell line samples. β-actin and histone H3 were used as the controls for loading and fractionation. **g** Morphological changes that occurred when LO2, HepG2, HepG2.2.15, and Hep3B cells were treated with TNF-α (40 ng/ml) for 4 h and observed under bright field microscopy. **h** LO2, HepG2 and Hep3B cells were treated with TNF-α (40 ng/ml) for 4 h, and the expression of ARRB1, p65 and p-p65 was analysed using western blotting. All values are the means ± SD of three separate experiments and were repeated three times. *P* < 0.01 or *P* < 0.05 using Student’s *t*-test

Our previous study showed that inflammatory TNF-α directly increases hepatic ARRB1 expression, resulting in malignant hepatocellular proliferation and carcinogenesis. Spontaneously, the relationship between ARRB1 and p65 in inflammation-induced hepatocarcinogenesis aroused our interest. To determine the critical residues of p-p65 in ARRB1-mediated hepatocellular carcinogenesis, ARRB1 expression was upregulated in HepG2 and Hep3B cells by a recombinant lentivirus expressing ARRB1. We found that p65 Ser536, but not Ser276 or Ser529, was markedly phosphorylated after ARRB1 overexpression in HepG2 and Hep3B cells (Fig. 5a). These results indicated that Ser536 was the critical residue of p-p65 in ARRB1-mediated hepatocellular carcinoma. To evaluate whether ARRB1 had any effect on p65 Ser536 in HCC cells, ARRB1 expression was upregulated in LO2, HepG2, HepG2.2.15 and Hep3B cells by a recombinant lentivirus expressing ARRB1 (Fig. 5b). We found that p65 Ser536 was markedly phosphorylated, while the total amounts of p65 remained the same as those in control cells (Fig. 5c, d). In response to a range of stimuli, phosphorylated cytoplasmic p65 relocates to the nucleus and promotes the activation of a series of target genes. An increase in the fluorescence intensity of p65 in the nucleus was confirmed using immunofluorescence (Fig. 5e). To further confirm that ARRB1 facilitates p65 translocation into the nucleus, nuclear and cytoplasmic fractions were isolated and examined from LO2, HepG2, and Hep3B cell line samples after ARRB1 transfection. The results showed increased p65 nuclear translocation after ARRB1 transfection (Fig. 5f). We then treated three HCC cell lines with TNF-α (40 ng/ml), and markedly increased ARRB1 expression was observed, as confirmed by our previous study [16], while p-p65 levels were found to be elevated in HCC cell lines compared with the control TNF-α-treated cells (Fig. 5g, h). These results indicated that the phosphorylation of p65 could be upregulated by the inflammation-mediated ARRB1 pathway in vitro.

### Phosphorylation of NF-κBp65 enhanced hepatocellular proliferation through the Akt/mTOR pathway in response to inflammation

**Fig. 6:**
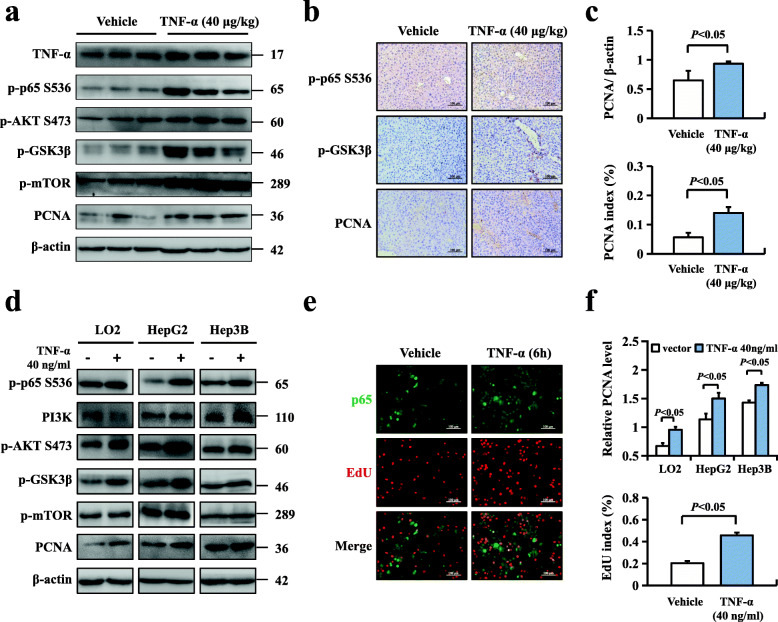
Phosphorylation of NF-κBp65 enhanced hepatocellular proliferation through the Akt/mTOR pathway in response to inflammation. **a** WT mice were intraperitoneally injected with TNF-α (40 μg/kg) for 6 h, and TNF-α-induced inflammation upregulated the protein expression of ARRB1, p-Akt, p-GSK-3β, m-TOR and PCNA in mouse livers, as shown by western blotting. **b** Representative images of p-p65 (top), p-GSK-3β (middle) and PCNA (bottom) staining in TNF-α-induced liver inflammation. **c** The relative PCNA protein level and PCNA index were scored (*n* = 6 in each group). All values are mean ± SD. *P* < 0.05 using Student’s *t*-test. **d** LO2, HepG2 and Hep3B cells were treated with TNF-α (40 ng/ml) for 6 h, and the expression of ARRB1 was analysed by western blotting. TNF-α induced p-p65 expression and upregulated the protein expression of p-Akt, p-GSK-3β, m-TOR and PCNA in cell lines using western blotting. **e** Images showing double staining of p-p65 and EdU in TNF-α (40 ng/ml)-pretreated HepG2 cells. Shown is a representative result of three experiments. **f** The relative PCNA protein level and EdU index were scored. All values are mean ± SD. *P* < 0.05 using Student’s *t*-test

Accumulating evidence has found that the Akt/GSK3β pathway can accelerate cell cycle progression in various types of tumours, and a previous study reported that Akt signalling could promote survival or inhibit apoptosis through substrates mediated in proportion by NF-κB [24]. It remains unknown whether the Akt/GSK3β signalling pathway is related to the phosphorylation of p65 during HCC cell proliferation. To explore the possible mechanism of p-p65 in hepatocarcinogenesis, we treated WT mice with TNF-α (40 μg/kg) and found significantly increased protein levels of p-p65, p-Akt, PCNA and downstream components of Akt, including p-GSK3β and m-TOR, in mouse livers using western blotting and obviously increased expression of p-p65 and PCNA using immunohistochemistry (Fig. 6a-c). We also observed that pretreatment with TNF-α (40 ng/ml) for 6 h promoted the expression of ARRB1 and p-p65 in HCC cell lines and found that p-Akt, p-GSK3β, p-mTOR and PCNA were markedly increased, consistent with the data obtained from WT mice. HCC cell proliferation was confirmed to be upregulated after TNF-α pretreatment of HepG2 cells, represented by double staining of p-p65 and EdU (Fig. 6d-f). Altogether, these results suggested that phosphorylation of NF-κBp65 enhanced hepatocellular proliferation through the GSK3β/mTOR pathway.

### Phosphorylation of NF-κBp65 induced GSK3β/mTOR signalling in an ARRB1-dependent manner

**Fig. 7:**
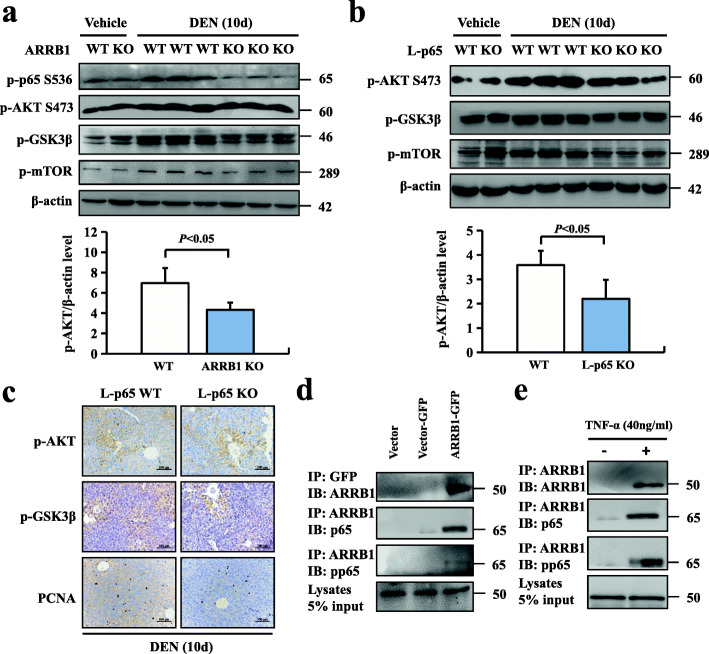
Phosphorylation of NF-κBp65 induced Akt/mTOR signalling in an ARRB1-dependent manner. **a**, **b** L-p65 KO mice and ARRB1-KO mice were treated with either physiological saline (vehicle) or 100 mg/kg DEN intraperitoneal injection for 10 days. The protein levels of ARRB1, p-p65, p-Akt p-GSK-3 and m-TOR. Relative p-p65 level was scored (*n* = 6 in each group). All values are mean ± SD. *P* < 0.05 using Student’s *t*-test. (*n* = 6 in each group). **c** Representative images of p-p65 (top), p-GSK3β (middle) and PCNA (bottom) staining in DEN-induced L-p65 KO mouse liver inflammation. **d** ARRB1-overexpressing cells were established as described. The cellular lysates of ARRB1-transfected HepG2 cells were subjected to immunoprecipitation with an anti-GFP antibody. Coimmunoprecipitated endogenous p65 and p-p65 were detected. **e** TNF-α (40 ng/ml)-treated HepG2 cell lysates were subjected to immunoprecipitation with an anti-ARRB1 antibody. Coimmunoprecipitated endogenous p65 and p-p65 were detected with anti-p65 or p-p65 antibodies. Shown is a representative result of three experiments. IP = immunoprecipitated; IB = immunoblot

Although previous results have suggested that ARRB1 can promote the phosphorylation of p65 in vivo and that phosphorylation of p65 promotes HCC cell proliferation through the GSK3β/mTOR pathway, it remains unknown whether the p65/GSK3β/mTOR signalling pathway is ARRB1 dependent in DEN-induced hepatocellular carcinogenesis. We evaluated Akt, p65 and GSK3β phosphorylation in both ARRB1 WT and ARRB1 KO mice after DEN treatment for 10 days. ARRB1 deficiency significantly weakened DEN-induced Akt, p65, GSK3β, m-TOR phosphorylation and PCNA expression (Fig. 7a). We then examined Akt and GSK3β phosphorylation in both WT and *L-p65* KO mice after DEN treatment for 10 days, and our data showed that p65 deficiency significantly weakened DEN-induced GSK3β, m-TOR phosphorylation and PCNA expression but did not affect the phosphorylation of Akt (Fig. 7b) or increase the expression of p-GSK3β and PCNA using immunohistochemistry (Fig. 7c). The preceding results suggested that ARRB1 enhanced the phosphorylation of p65 and induced hepatocellular proliferation, but whether ARRB1 and p65 directly interact (including p-p65) remains unclear. We then performed a coimmunoprecipitation assay in ARRB1-overexpressing HepG2 cells and found that both p-p65 and p65 could directly bind to ARRB1 (Fig. 7d). Furthermore, TNF-α significantly increased the interaction of ARRB1 with p65 and p-p65 (Fig. 7e). Considered together, our results indicated that phosphorylation of NF-κBp65 induced hepatocellular carcinogenesis through ARRB1-dependent GSK3β/mTOR signalling.

### Akt/mTOR signalling, and the *ARRB1* promoter were activated through NF-κBp65 phosphorylation on Ser536

**Fig. 8:**
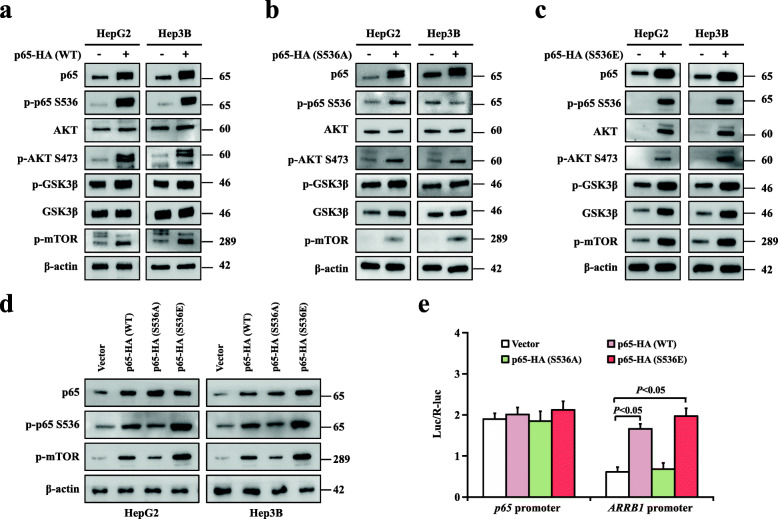
Akt/mTOR signalling, and the *ARRB1* promoter were activated through ARRB1-mediated NF-κBp65 phosphorylation at Ser536. **a** NF-κBp65 overexpression increased the expression of p-Akt, p-GSK3β and p-m-TOR in HepG2 and HepG3B cell lines. **b**, **c** p65 Ser536A (inactive) overexpression slightly increased, while p65 Ser536E (active) overexpression resulted in arrested increases in the expression of p-Akt, p-GSK3β and p-m-TOR in HepG2 and HepG3B cell lines. **d** p65 Ser536E (active) overexpression significantly increased the expression of p-m-TOR in HepG2 and HepG3B cell lines. **e** p65 Ser536A (inactive) overexpression slightly increased, while p65 Ser536E (active) overexpression obviously increased the activity of *ARRB1* promoter, but not the *p65* promoter, in 293 T cells. All values are mean ± SD. *P* < 0.05 using Student’s *t*-test

Our data proved that Ser536 was the critical residue of p-p65 in ARRB1-mediated hepatocellular carcinogenesis (Fig. 5a). Herein, we overexpressed normal active (WT), constitutively inactive (Ser536A), and constitutively active (Ser536E) NF-κBp65 in HepG2 and Hep3B cells. We found significantly increased levels of p-p65, p-Akt, p-GSK3β, and especial the p-m-TOR level, in active p65 Ser536E cells and, conversely, less increased levels of p-p65, p-Akt, p-GSK3β and p-m-TOR in inactive p65 Ser536A cells (Fig. 8a-d). Considering that both p65 and ARRB1 were upregulated in HCC, we further examined the activity of the *p65* promoter and *ARRB1* promoter after overexpressing normal active (WT), constitutively inactive (Ser536A), and constitutively active (Ser536E) NF-κBp65 in 293 T cells. Increased activities of the *ARRB1* promoter, but not the *p65* promoter, was observed in active p65 Ser536E cells, while conversely, less increased activities of the *ARRB1* promoter was observed in inactive p65 Ser536A cells (Fig. 8e). Altogether, we confirmed that inflammation mediated hepatocellular carcinogenesis through phosphorylation of NF-κBp65 on Ser536.

## Discussion

A number of studies have examined the molecular mechanisms that connect inflammation to tumorigenesis, and NF-κB is a group of transcription factors that play central roles in inflammation. ARRB1 can promote HCC cell proliferation through the PI3K/Akt pathway and function as an enhancer of the GPCR-stimulated NF-κB pathway. Considering that NF-κBp65 was found to be related to the progression of HCC, we revealed that p65 and phosphorylation of p65 are upregulated in HCC patients and essential to mouse acute hepatitis and liver cancer. Our data suggested that inflammation induced proinflammatory cytokines and enhanced the expression of ARRB1, which activated the phosphorylation of NF-κBp65, resulting in hepatocarcinogenesis by hepatocellular compensatory proliferation [25, 26].

This observation is consistent with the earliest report of NF-κB in carcinogenesis by multidrug resistance protein 2 (MDR2) ­deficient (Mdr2^−/−^) mice, which were found to spontaneously develop cholestatic hepatitis and HCC. The inhibition of NF-κB in Mdr2^−/−^ mice, obtained from the introduction of nondegradable IκB, blocked the progression of hepatocellular carcinoma by hepatocyte apoptosis [27]. Another study deleted IKKβ in intestinal epithelial cells and found a significant decrease in tumour incidence [8]. The IKKβ (EE)^Hep^ model, with constitutive IKK activation, showed apparent ectopic lymphoid-like structures (ELSs), which promoted the progression of hepatocarcinogenesis both spontaneously and through DEN treatment through a mechanism that provided crucial survival and growth factors from the immune microniche environment [28]. In contrast, subsequent studies suggested that IKKβ-deficient mice had markedly increased hepatocarcinogenesis after DEN treatment by compensatory proliferation, and hepatocyte-specific ablation of IKKγ/NEMO mice was found to spontaneously result in steatohepatitis followed by hepatocellular carcinoma [26, 29, 30]. However, a previous study determined the different responses to TNF treatment in hepatocyte-deficient IKKβ mice and hepatocyte RelA/p65-deficient mice caused by residual NF-κB activity [31]. IKKβ-deficient hepatocytes were mildly sensitive to TNF-induced apoptosis, but RelA/p65-deficient hepatocytes were highly sensitive to TNF treatment. In this context, mice with conditional ablation of RelA/p65, IKKα/IKKβ or IKKγ/NEMO efficiently inhibited the NF-κB pathway but exhibited different hepatic phenotypes after treatment with TNF. More recent results have found that the controversy might be caused by NF-κB-independent functions [6, 23, 32]. A follow-up study suggested that IKKα/IKKβ-dependent phosphorylation of receptor-interacting kinases (RIPKs) decreased the promotion of spontaneous liver tumours by inhibiting hepatocyte compensatory proliferation [33]. Furthermore, obesity and insulin resistance increased the risk of liver cancers, and our previous study suggested that inactivation of p65 in mouse livers improved insulin sensitivity through the cAMP/PKA pathway [21, 34]. Together, these results were consistent with our results and confirmed the decreased tumour incidence after DEN treatment in L-p65-KO mice.

PI3K/Akt not only leads to the activation of both GSK3β and mTOR but also has been found upstream of NF-κB activation in different cancers, and the NF-κB pathway is believed to be a target of Akt [24, 35, 36]. Here, we identified that phosphorylation of p65 enhanced hepatocarcinogenesis through the GSK-3β/mTOR pathway. A recent study confirmed our results and suggested that inhibition of GSK3β decreased p-mTOR levels and downstream molecules, which were related to cell survival and growth in HCC cells and inhibited tumour growth in vivo [20].

Previous studies have found that ARRB1 enhances hepatocarcinogenesis by inflammation-mediated signalling but inhibits the activation of NF-κB [16, 37]. However, a recent view of the GPCR field has demonstrated that GPCR signalling can be directly transduced through ARRB1 and that the translocation of ARRB1 to the nucleus enhances NF-κB activity [38–40], consistent with our findings that overexpression of ARRB1 in HCC cell lines increases the level of p65 phosphorylation and promotes p65 translocation into the nucleus. Phosphorylation of p65 at Ser536 plays a vital role in hepatocellular carcinogenesis [41]. In this study, our results showed that ARRB1 significantly induces the phosphorylation of p65 Ser536 in human HCC cell lines and that phosphorylation of p65 Ser536 is involved in ARRB1-mediated hepatocarcinogenesis.

Other studies have shown that ARRB1 regulates several cancer-related cellular processes via diverse signal transduction pathways, including the Ras/Raf/MEK/ERK family, β-catenin/TCF4 signalling, Akt/GSK3β signalling, mTOR signalling and NF-κB signalling [38]. We then investigated the downstream molecular mechanisms of ARRB1-induced NF-κBp65 activation in hepatocellular carcinogenesis. The results showed that ARRB1 could interact with p65 Ser536, and the interaction was promoted after TNF-α treatment. The activities of Akt/mTOR signalling, the *ARRB1* promoter was NF-κBp65 Ser536 dependent, confirming the important role of ARRB1 in the NF-κBp65 Ser536 pathway.

## Conclusions

**Fig. 9:**
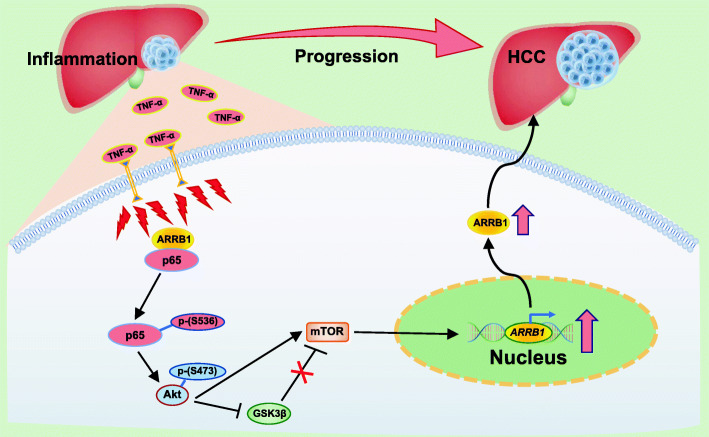
Diagram of the mechanism by which NF-κBp65 phosphorylation drives hepatocellular carcinogenesis. Inflammation, such as TNF-α, induces ARRB1 and promotes ARRB1 binding to NF-κBp65, facilitating the phosphorylation of p65 at ser536. Then, the phosphorylation of p65 (Ser536) activates Akt/mTOR signalling to drive malignant hepatocellular proliferation, resulting in hepatocellular carcinogenesis

In conclusion, ARRB1 induced hepatocellular carcinogenesis via interaction with p65 to promote malignant hepatocellular proliferation through the GSK3β/mTOR pathway (Fig. 9). Thus, inhibition of phosphorylation of NF-κBp65 at Ser536 has therapeutic benefits, and be targets of new pharmacotherapies for HCC patients.

## Supplementary Information

**Additional file 1: Figure S1.** Deficiency of hepatocytes p65 attenuated hepatocellular carcinogenesis in mice. **(a)** AFP levels of WT and L-p65-KO mice. **(b)** Quantification of average liver weight versus body weight in WT and L-p65-KO mice. **(c)** Maximal size of liver tumors measured by a caliper. **(d)** Average tumor numbers at 9 months. All values are mean ± SD (*n* = 10 for each group). *P* < 0.05 by using Student’s *t*-test.

**Additional file 2: Figure S2.** Cell proliferation was increased in DEN-induced HCC mouse model. **(a)** Representative images of ki67 staining in DEN-induced HCC. **(b)** PCNA expression in mice liver was determined by western blotting. **(c)** Ki67 index and PCNA relative protein level was scored. All values are mean ± SD (*n* = 6 in each group). *P* < 0.05 by using Student’s *t*-test.

**Additional file 3: Figure S3.** The mRNA levels of *p65* and *TNF-α* in the acute inflammatory mouse models were measured by Real time-PCR. **(a)** The mRNA level of *p65* was elevated in liver tissues after intraperitoneal injection of CCl_4_ for 2 months, DEN for 10 days, or TNF-α (40 μg/kg) for 6 h. **(b)** The *TNF-α* mRNA level was elevated in liver tissues after intraperitoneal injection of CCl_4_ for 2 months, DEN for 10 days, or TNF-α (40 μg/kg) for 6 h. All values are mean ± SD (*n* = 6 in each group). *P* < 0.05 by using Student’s *t*-test.

## Data Availability

The data in the current study are available from the corresponding authors upon reasonable request.

## Abbreviations

NF-κB: Nuclear factor­κB
ARRB1: β-arrestin1
HCC: Hepatocellular carcinoma
EMT: Epithelial-mesenchymal transition
GPCRs: G protein-coupled receptors
TNF: Tumour necrosis factor
RHD: Rel homology domain
WT: Wild type
DEN: Diethylnitrosamine
CCl_4_: Carbon tetrachloride
NLS: Nuclear localization signal
RIPKs: Receptor-interacting kinases
MDR2: Multidrug resistance protein 2
ELSs: Ectopic lymphoid-like structures
