# Supplementary material for: Phosphorylation of NF-κBp65 drives inflammation-mediated hepatocellular carcinogenesis and is a novel therapeutic target
Source: J Exp Clin Cancer Res. 2021 Aug 11;40:253. doi: 10.1186/s13046-021-02062-x (PMC8359590; doi:10.1186/s13046-021-02062-x)
Supplement: Supplementary file 1 — Additional file 1: Figure S1. Deficiency of hepatocytes p65 attenuated hepatocellular carcinogenesis in mice. (a) AFP levels of WT and L-p65-KO mice. (b) Quantification of average liver weight versus body weight in WT and L-p65-KO mice. (c) Maximal size of liver tumors measured by a caliper. (d) Average tumor numbers at 9 months. All values are mean ± SD (n = 10 for each group). P < 0.05 by using Student’s t-test. [file 13046_2021_2062_MOESM1_ESM.pdf]

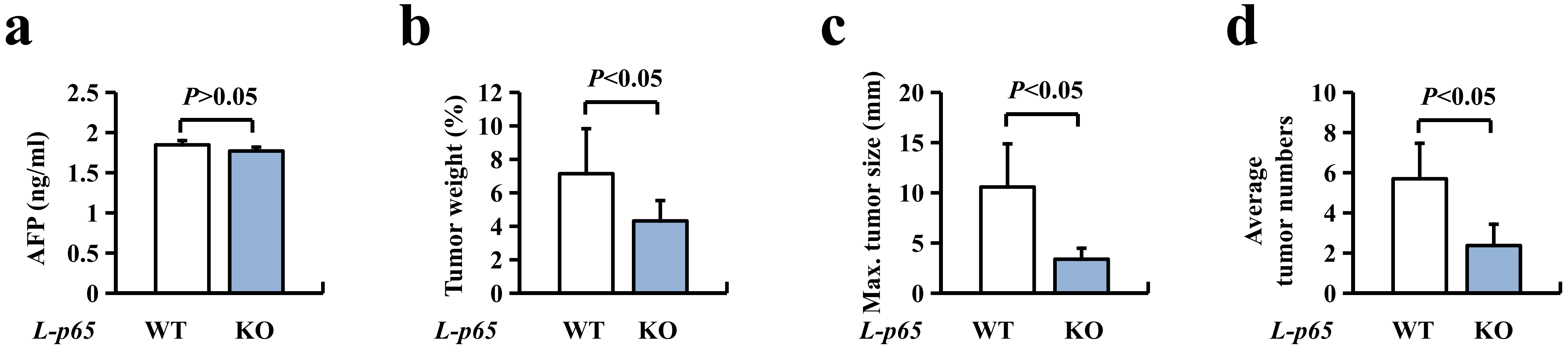

Fig. S1 Deficiency of hepatocytes p65 attenuated hepatocellular carcinogenesis in mice. (a) AFP levels of WT and L-p65-KO mice. **(b)** Quantification of average liver weight versus body weight in WT and L-p65-KO mice. **(c)** Maximal size of liver tumors measured by a caliper. **(d)** Average tumor numbers at 9 months . All values are mean ± SD (n=10 for each group). *P*<0.05 by using Student's *t*-test.
