# Supplementary material for: Phosphorylation of NF-κBp65 drives inflammation-mediated hepatocellular carcinogenesis and is a novel therapeutic target
Source: J Exp Clin Cancer Res. 2021 Aug 11;40:253. doi: 10.1186/s13046-021-02062-x (PMC8359590; doi:10.1186/s13046-021-02062-x)
Supplement: Supplementary file 2 — Additional file 2: Figure S2. Cell proliferation was increased in DEN-induced HCC mouse model. (a) Representative images of ki67 staining in DEN-induced HCC. (b) PCNA expression in mice liver was determined by western blotting. (c) Ki67 index and PCNA relative protein level was scored. All values are mean ± SD (n = 6 in each group). P < 0.05 by using Student’s t-test. [file 13046_2021_2062_MOESM2_ESM.pdf]

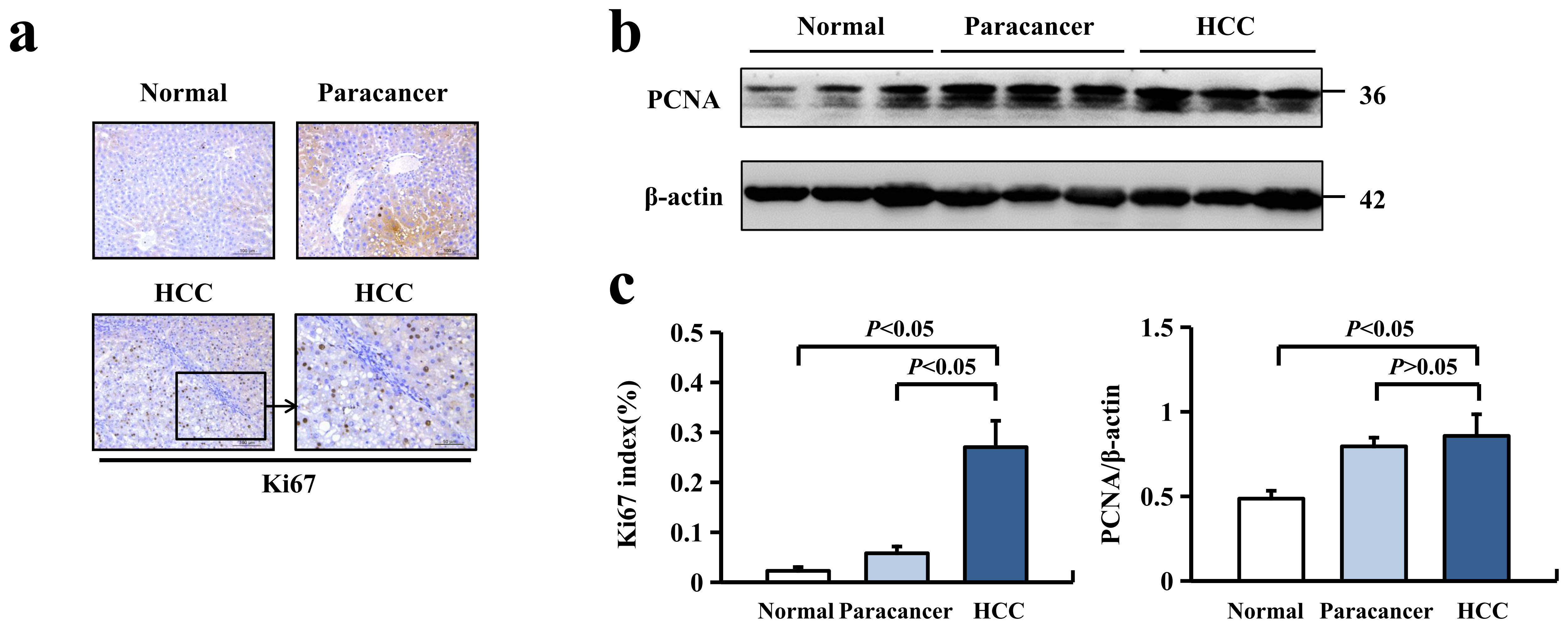

Fig. S2 Cell proliferation was increased in DEN-induced HCC mouse model. (a) Representative images of ki67 staining in DEN-induced HCC. **(b)** PCNA expression in mice liver was determined by western blotting. **(c)** Ki67 index and PCNA relative protein level was scored. All values are mean ± SD (n=6 in each group). *P*<0.05 by using Student's *t*-test.
