# Supplementary material for: Phosphorylation of NF-κBp65 drives inflammation-mediated hepatocellular carcinogenesis and is a novel therapeutic target
Source: J Exp Clin Cancer Res. 2021 Aug 11;40:253. doi: 10.1186/s13046-021-02062-x (PMC8359590; doi:10.1186/s13046-021-02062-x)
Supplement: Supplementary file 3 — Additional file 3: Figure S3. The mRNA levels of p65 and TNF-α in the acute inflammatory mouse models were measured by Real time-PCR. (a) The mRNA level of p65 was elevated in liver tissues after intraperitoneal injection of CCl4 for 2 months, DEN for 10 days, or TNF-α (40 μg/kg) for 6 h. (b) The TNF-α mRNA level was elevated in liver tissues after intraperitoneal injection of CCl4 for 2 months, DEN for 10 days, or TNF-α (40 μg/kg) for 6 h. All values are mean ± SD (n = 6 in each group). P < 0.05 by using Student’s t-test. [file 13046_2021_2062_MOESM3_ESM.pdf]

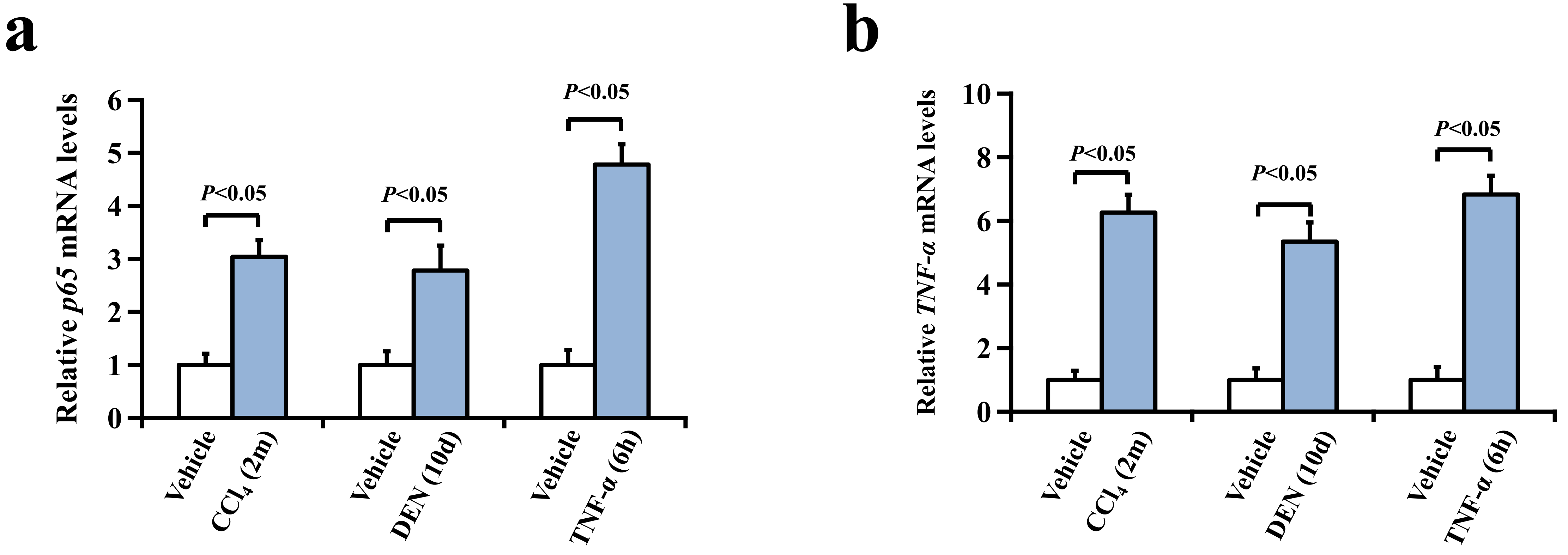

Fig. S3 The mRNA levels of *p65* and *TNF-α* in the acute inflammatory mouse models were measured by Real time-PCR. (a) The mRNA level of *p65* was elevated in liver tissues after intraperitoneal injection of CCl_4 for 2 months, DEN for 10 days, or TNF-α (40 μg/kg) for 6h. **(b)** The *TNF-α* mRNA level was elevated in liver tissues after intraperitoneal injection of CCl_4 for 2 months, DEN for 10 days, or TNF-α (40 μg/kg) for 6h. All values are mean ± SD (n=6 in each group). $P<0.05$ by using Student's *t*-test.
